# Tuberculosis treatment outcomes and patient support groups, southern India

**DOI:** 10.2471/BLT.22.288237

**Published:** 2022-11-15

**Authors:** Rajaram Subramanian Potty, Karthikeyan Kumarasamy, Joseph F Munjattu, Ramesh C Reddy, Rajesham Adepu, Anil Singarajipura, Mohan H Lakkappa, Reuben Swamickan, Amar Shah, Vikas Panibatla, Reynold Washington

**Affiliations:** a Karnataka Health Promotion Trust (KHPT), IT Park, Rajajinagar Industrial Area, Behind KSSIDC Administration Office, Rajajinagar, Bengaluru, Karnataka, India 560044.; b Office of the Joint Director, Lady Willingdon State TB Centre, Bengaluru, India.; c Office of the Joint Director, Commissionerate of Health and Family Welfare, Hyderabad, India.; d Tuberculosis and Infectious Diseases Division, United States Agency for International Development India, New Delhi, India.; e TB Alert India, Hyderabad, India.; f St John’s Research Institute, Bengaluru, India.

## Abstract

**Objective:**

To assess treatment outcomes in tuberculosis patients participating in support group meetings in five districts of Karnataka and Telangana states in southern India.

**Methods:**

Tuberculosis patients from five selected districts who began treatment in 2019 were offered regular monthly support group meetings, with a focus on patients in urban slum areas with risk factors for adverse outcomes. We tracked the patients’ participation in these meetings and extracted treatment outcomes from the Nikshay national tuberculosis database for the same patients in 2021. We compared treatment outcomes based on attendance of the support groups meetings.

**Findings:**

Of 30 706 tuberculosis patients who started treatment in 2019, 3651 (11.9%) attended support groups meetings. Of patients who attended at least one support meeting, 94.1% (3426/3639) had successful treatment outcomes versus 88.2% (23 745/26 922) of patients who did not attend meetings (adjusted odds ratio, aOR: 2.44; 95% confidence interval, CI: 2.10–2.82). The odds of successful treatment outcomes were higher in meeting participants than non-participants for all variables examined including: age ≥ 60 years (aOR: 3.19; 95% CI: 2.26–4.51); female sex (aOR: 3.33; 95% CI: 2.46–4.50); diabetes comorbidity (aOR: 3.03; 95% CI: 1.91–4.81); human immunodeficiency virus infection (aOR: 3.73; 95% CI: 1.76–7.93); tuberculosis retreatment (aOR: 1.69; 1.22–2.33); and drug-resistant tuberculosis (aOR: 1.93; 95% CI: 1.21–3.09).

**Conclusion:**

Participation in support groups for tuberculosis patients was significantly associated with successful tuberculosis treatment outcomes, especially among high-risk groups. Expanding access to support groups could improve tuberculosis treatment outcomes at the population level.

## Résumé

**Objectif:**

Évaluer les résultats des traitements contre la tuberculose chez les patients qui assistent aux réunions des groupes d'entraide dans cinq districts des États du Karnataka et du Telangana, en Inde du Sud.

**Méthodes:**

Dans les cinq districts sélectionnés, des patients tuberculeux ayant entamé un traitement en 2019 se sont vu proposer des réunions mensuelles organisées à intervalles réguliers. Ces réunions étaient principalement destinées aux patients vivant dans des bidonvilles urbains et présentant des facteurs augmentant le risque d'issue défavorable. Nous avons suivi leur participation aux réunions et avons extrait des informations thérapeutiques de la base de données nationale Nikshay sur la tuberculose concernant les mêmes patients en 2021. Nous avons ensuite comparé l'issue des traitements à la présence aux réunions des groupes d'entraide.

**Résultats:**

Sur 30 706 patients tuberculeux ayant entamé un traitement en 2019, 3651 (11,9%) ont rejoint des groupes d'entraide. Sur l'ensemble des patients ayant assisté à au moins une réunion, 94,1% (3426/3639) ont connu une issue favorable au traitement, contre 88,2% (23 745/26 922) chez les patients qui n'y ont pas assisté (odds ratio ajusté, ORA: 2,44; intervalle de confiance de 95%, IC: 2,10-2,82). Les probabilités de réussite du traitement étaient plus élevées chez les participants aux réunions que chez les non-participants pour toutes les variables examinées, y compris pour les ≥ 60 ans (ORA: 3,19; IC de 95%: 2,26–4,51); les patients de sexe féminin (ORA: 3,33; IC de 95%: 2,46–4,50); celles et ceux présentant une comorbidité liée au diabète (ORA: 3,03; IC de 95%: 1,91–4,81); une infection au virus de l'immunodéficience humaine (ORA: 3,73; IC de 95%: 1,76–7,93); un retraitement contre la tuberculose (ORA: 1,69; 1,22–2,33); et enfin, une tuberculose pharmacorésistante (ORA: 1,93; IC de 95%: 1,21–3,09).

**Conclusion:**

La participation des patients atteints de tuberculose aux groupes d'entraide allait de pair avec de meilleurs résultats de traitement, surtout au sein des catégories à haut risque. Promouvoir l'accès à ces groupes d'entraide pourrait améliorer l'issue des traitements contre la tuberculose à l'échelle de la population.

## Resumen

**Objetivo:**

Evaluar los desenlaces del tratamiento en pacientes con tuberculosis que participan en reuniones de grupos de apoyo en cinco distritos de los estados de Karnataka y Telangana en el sur de la India.

**Métodos:**

Se ofrecieron reuniones mensuales regulares de grupos de apoyo a los pacientes con tuberculosis de cinco distritos seleccionados que comenzaron el tratamiento en 2019, con un enfoque en los pacientes de los suburbios urbanos con factores de riesgo de desenlaces adversos. Se realizó un seguimiento de la participación de los pacientes en estas reuniones y se extrajeron los desenlaces del tratamiento de la base de datos nacional de tuberculosis Nikshay para los mismos pacientes en 2021. Se compararon los desenlaces del tratamiento en función de la asistencia a las reuniones de los grupos de apoyo.

**Resultados:**

De 30 706 pacientes con tuberculosis que iniciaron el tratamiento en 2019, 3651 (11,9 %) asistieron a reuniones de grupos de apoyo. De los pacientes que asistieron al menos a una reunión de apoyo, el 94,1 % (3426/3639) presentaron desenlaces exitosos del tratamiento frente al 88,2 % (23 745/26 922) de los pacientes que no asistieron a las reuniones (razón de posibilidades ajustada, RPA: 2,44; intervalo de confianza del 95 %, IC: 2,10-2,82). Las posibilidades de obtener un desenlace satisfactorio del tratamiento fueron mayores en los participantes en las reuniones que en los no participantes para todas las variables examinadas, incluyendo: edad ≥60 años (RPA: 3,19; IC del 95 %: 2,26-4,51); sexo femenino (RPA: 3,33; IC del 95 %: 2,46-4,50); comorbilidad por diabetes (RPA: 3,03; IC del 95 %: 1,91-4,81); infección por el virus de la inmunodeficiencia humana (RPA: 3,73; IC del 95 %: 1,76-7,93); retratamiento de la tuberculosis (RPA: 1,69; IC del 95 %: 1,22-2,33); y tuberculosis resistente (RPA: 1,93; IC del 95 %: 1,21-3,09).

**Conclusión:**

La participación en grupos de apoyo para pacientes con tuberculosis se asoció de manera significativa con el éxito de los desenlaces del tratamiento de la tuberculosis, en especial entre los grupos de alto riesgo. Ampliar el acceso a los grupos de apoyo podría mejorar los desenlaces del tratamiento de la tuberculosis a nivel de la población.

## ملخص

**الغرض:**

تقييم نتائج العلاج في مرضى السل المشاركين في اجتماعات مجموعة الدعم، في خمس مقاطعات من ولايتي كارناتاكا وتيلانجانا في جنوب الهند.

**الطريقة:**

تم عرض اجتماعات مجموعة دعم شهرية منتظمة على مرضى السل من خمس مقاطعات محددة، وكان المرضى قد بدأوا العلاج في عام 2019، مع التركيز على المرضى في منطقة الأحياء الفقيرة الحضرية مع عوامل الخطر لنتائج سلبية. قمنا بتتبع مشاركة المرضى في هذه الاجتماعات، واستخلصنا نتائج العلاج من قاعدة بيانات السل الوطنية في Nikshay لنفس المرضى في عام 2021. قمنا بمقارنة نتائج العلاج بناءً على حضور اجتماعات مجموعات الدعم.

**النتائج:**

من بين 30706 مريضًا بالسل بدأوا العلاج في عام 2019، حضر 3651 مريضًا (11.9%) اجتماعات مجموعات الدعم. من بين المرضى الذين حضروا اجتماع دعم واحد على الأقل، حقق 94.1% (3426/3639) نتائج علاجية ناجحة، مقابل 88.2% (23745/26922) من المرضى الذين لم يحضروا الاجتماعات (نسبة الاحتمالات المعدلة: 2.44؛ بفاصل ثقة مقداره 95%: 2.10 إلى 2.82). كانت احتمالات نتائج العلاج الناجحة أعلى المشاركين بالاجتماعات مقارنة بغير المشاركين لجميع المتغيرات التي تم فحصها بما في ذلك: العمر أكبر من أو يعادل 60 عامًا (نسبة الاحتمالات المعدلة 3.19؛ ‏بفاصل ثقة مقداره 95%: 2.26 إلى 4.51)؛ والنسبة للإناث (نسبة الاحتمالات المعُدلة: 3.33؛ ‏بفاصل ثقة مقداره 95%: 2.46 إلى 4.50)؛ ولأمراض المصاحبة لمرض السكري (نسبة الاحتمالات المعُدلة: 3.03؛ ‏بفاصل ثقة مقداره 95%: 1.91 إلى 4.81)؛ وعدوى فيروس نقص المناعة البشرية (نسبة الاحتمالات المعُدلة: 3.73؛ ‏بفاصل ثقة مقداره 95%: 1.76 إلى 7.93)؛ وعلاج السل (نسبة الاحتمالات المعُدلة: 1.69؛ 1.22 إلى 2.33)؛ والسل المقاوم للأدوية (نسبة الاحتمالات المعُدلة: 1.93؛ ‏بفاصل ثقة مقداره 95%: 1.21 إلى 3.09).

**الاستنتاج:**

ارتبطت المشاركة في مجموعات الدعم لمرضى السل بشكل كبير بنتائج علاج السل الناجحة، خاصة بين المجموعات المعرضة للخطر. يمكن لتوسيع الوصول إلى مجموعات الدعم أن يُحسّن من نتائج علاج السل على مستوى السكان.

## 摘要

**目的:**

旨在评估参加在印度南部卡纳塔克邦和特伦甘纳邦的五个县内举办的支持小组会议的结核病患者的治疗结果。

**方法:**

邀请五个选定县内自 2019 年起开始接受治疗的结核病患者每月定期参加支持小组会议，重点关注城市贫民区内存在不良后果风险因素的患者。2021 年，我们跟踪了患者参与这些会议的情况，并从 Nikshay 国家结核病数据库中提取了这些患者的治疗结果。我们根据支持小组会议的参与情况比较了治疗结果。

**结果:**

在自 2019 年起开始接受治疗的 30,706 名结核病患者中，3651 人（占 11.9%）参加了支持小组会议。至少参加过一次支持会议的患者的治疗结果成功率为 94.1% (3426/3639)，相比之下，从未参与会议的患者的治疗结果成功率为 88.2% (23,745/26,922)（调整后优势比，aOR：2.44；95％ 置信区间，CI: 2.10–2.82）。基于所有检查变量，参与会议的患者的治疗结果成功率均高于未参与会议的患者，包括：年龄≥ 60 岁 (aOR: 3.19; 95% CI: 2.26–4.51)；女性 (aOR: 3.33; 95% CI: 2.46–4.50)；糖尿病合并症 (aOR: 3.03; 95% CI: 1.91–4.81)；人类免疫缺陷病毒感染 (aOR: 3.73; 95% CI: 1.76–7.93)；结核病再治疗 (aOR: 1.69; 1.22–2.33)；以及耐多药结核病 (aOR: 1.93; 95% CI: 1.21-3.09）。

**结论:**

参与结核病患者支持小组与结核病治疗结果成功率存在明显相关性，特别是对于高危人群。提供更多支持小组可以全面提高结核病患者的治疗结果成功率。

## Резюме

**Цель:**

Оценить результаты лечения у больных туберкулезом, участвующих в собраниях групп поддержки в пяти районах штатов Карнатака и Телангана на юге Индии.

**Методы:**

Больным туберкулезом из пяти выбранных районов, начавшим лечение в 2019 году, были предложены регулярные ежемесячные встречи группы поддержки с особым вниманием к пациентам из городских трущоб с факторами риска развития неблагоприятных исходов. Участие пациентов в этих встречах отслеживалось, и результаты лечения тех же пациентов в 2021  году были извлечены из национальной базы данных по туберкулезу Nikshay. Было проведено сравнение результатов лечения на основании посещаемости собраний групп поддержки.

**Результаты:**

Из 30 706 больных туберкулезом, начавших лечение в 2019 году, 3651 (11,9%) посещали встречи групп поддержки. Среди пациентов, посетивших хотя бы одну встречу поддержки, у 94,1% (3426/3639) наблюдались успешные результаты лечения по сравнению с 88,2% (23 745/26 922) пациентов, не посещавших встречи (скорректированное отношение шансов, сОШ: 2,44; 95%-й ДИ: 2,10–2,82). Шансы на успешный исход лечения были выше у участников встреч по сравнению с теми, кто не принимал в них участия, по всем исследованным переменным, включая возраст ≥ 60 лет (сОШ: 3,19; 95%-й ДИ: 2,26–4,51), женский пол (сОШ: 3,33; 95%-й ДИ: 2,46–4,50), коморбидную патологию на фоне сахарного диабета (сОШ: 3,03; 95%-й ДИ: 1,91–4,81), инфицирование вирусом иммунодефицита человека (сОШ: 3,73; 95%-й ДИ: 1,76–7,93), повторное лечение туберкулеза (сОШ: 1,69; 95%-й ДИ: 1,22–2,33) и лекарственно-устойчивый туберкулез (сОШ: 1,93; 95%-й ДИ: 1,21–3,09).

**Вывод:**

Участие в группах поддержки для больных туберкулезом было в значительной степени связано с успешными результатами лечения туберкулеза, особенно среди групп с высоким риском. Расширение доступа к группам поддержки может способствовать улучшению результатов лечения туберкулеза на уровне населения.

## Introduction

Estimates indicate that India has the largest number of tuberculosis patients (26%) and tuberculosis-related deaths (36%) in the world.^1^ India’s success in tackling tuberculosis is critical to achieving the global goal of ending tuberculosis by 2030. India’s national strategic plan on tuberculosis 2017–2025 envisages achieving a treatment success rate of 92% and 75% among individuals with drug-sensitive and drug-resistant tuberculosis, respectively, by 2025.^2^ Overall, the treatment success rate for drug-sensitive and drug-resistant tuberculosis was 81% (1 665 016/2 049 517) and 48% (16 668/34 621), respectively, in 2018.^3^ These figures highlight the need for highly effective and rapidly scalable interventions to accelerate the success rate in tuberculosis treatment outcomes.

Treatment approaches that include patients and their family members in a person-centred care process are more likely to be successful.^4^^,^^5^ Processes for empowering and involving tuberculosis patients in the prevention and control of their disease are of increasing interest to policy-makers, programme managers and health-care providers concerned with tuberculosis control.^6^ Many studies on other disease conditions show that empowering and involving patients is feasible using forums that facilitate sharing and learning from other patients’ experiences, challenges and successes.^6^^–^^12^ Peer support for tuberculosis patients has been attempted in many countries with varying degrees of success.^13^^–^^16^ In India, strategies for peer support and patient involvement have rarely been implemented and studied. In this paper, we describe implementation of support group meetings for tuberculosis patients and assess tuberculosis treatment outcomes in patients participating in these meetings.

## Methods

### Study setting

The Tuberculosis Health Action Learning Initiative, funded by the United States Agency for International Development, was implemented in selected districts of two states in southern India during 2016–2020. In total, 46 tuberculosis units from urban areas of Bellary, Bengaluru Urban and Koppal districts in Karnataka state and Warangal and Hyderabad districts in Telangana state were selected for the implementation of support group meetings in 2019. In consultation with state and district tuberculosis programme staff, districts with higher proportions of the urban population living in slums were selected.

### Implementation

We locally recruited community health workers (CHWs) from urban communities in 2016.^17^ In 2019, senior technical tuberculosis programme staff and technical staff of the project trained the CHWs and tuberculosis programme field staff to jointly organize and conduct monthly support group meetings within the public health facilities offering tuberculosis services. These health workers and programme staff formed support groups for tuberculosis patients who started treatment in 2019. Family members and/or caregivers of the patients and individuals who had previously completed tuberculosis treatment were included in the support groups. Tuberculosis patients who were very ill were excluded from participating. Support group members received information on the date and time of the meetings from CHWs or the tuberculosis programme staff. Initially, the health workers or programme staff provided this information during home or clinic visits. Later, district tuberculosis programme managers designated a specific day and time for the support group meetings and this information was stamped on the patient-held treatment card. The project provided CHWs and programme staff with a list of topics and behaviour change communication materials to facilitate the meetings, and they encouraged participants to actively interact during the meeting. Topics included stigma and disclosure, treatment adherence, nutrition, healthy living and protection of other family members from tuberculosis. Each meeting lasted for 60–90 minutes during which participants discussed only one topic. The groups consisted of 15–20 participants of mixed sex and age. Participants received no remuneration for participation in the meetings. Local donors supported the provision of light refreshments for the participants after the meeting. Tuberculosis programme staff formed additional patient support groups in settings where there were a large number of patients. Attendance of the meetings was voluntary and the CHWs and programme staff did not pressure the tuberculosis patients to attend a specific number of meetings during their treatment. The main intention was that each patient would attend the support group meeting once a month until they completed their treatment course. The CHWs recorded the attendance of the patients in each meeting with the Nikshay unique identification number, and they contacted patients who missed a meeting, either by telephone or personal visit, to maintain the number of participants. Nikshay is the national web-based database for tuberculosis patients developed by the Central Tuberculosis Division. Every tuberculosis patient notified has a unique patient identification number in Nikshay. Details of the support meetings can be found elsewhere.^18^^,^^19^

### Analyses

We used quantitative methods to examine the treatment outcomes of patients who attended and did not attend the support group meetings. Our primary record was the meeting register in which the patient’s name, age, sex and Nikshay identification number were recorded with the date and place of the meeting. We combined these data with the Nikshay database and removed duplicate entries based on the Nikshay identification number. The Nikshay database contains information on patient characteristics including: district of residence; type of tuberculosis; site of disease; alcohol use; comorbidity with diabetes and human immunodeficiency virus (HIV); and treatment outcomes. We analysed participation in support groups and successful treatment outcomes as the intervention outcomes. In line with national guidelines, we defined successful treatment outcome as cured (negative smear or culture at the end of the completion of treatment) or completed treatment. We report descriptive statistics with frequencies and percentages. We used logistic regression analysis to assess the association between participation in the support group meeting and successful treatment outcomes adjusted for various patient characteristics: age, sex, district of residence, type of tuberculosis case, disease site, comorbidities (diabetes and HIV infection) and regular alcohol consumption. We report unadjusted odds ratios (OR) and adjusted OR (aOR) and 95% confidence intervals (CI) of successful treatment outcomes. We used Stata version 14.0 (StataCorp LLC, College Station, United States of America) for the analysis.

### Ethics

The Institutional Ethics Committee of St John’s Medical College and Hospital, Bengaluru (study reference no. 187/2019) and the state tuberculosis offices in the two states provided ethics and regulatory approvals for conducting the study.

## Results

### Patient characteristics

In 2019, 30 706 tuberculosis patients started treatment in the study districts of Karnataka and Telangana states, of whom 3651 (11.9%) participated in the meetings. Of the 30 706 patients, 15 177 (49.4%) were from Hyderabad, 24 587 (80.1%) were in the economically productive age group of 15–59 years, 17 786 (57.9%) were males, 1801 (5.9%) had diabetes, 1146 (3.7%) had HIV infection, 1779 (5.8%) reported regular alcohol consumption, 20 987 (68.4%) had pulmonary tuberculosis, 26 733 (87.1%) were newly diagnosed, 2976 (9.7%) were being re-treated and 997 (3.3%) had drug-resistant tuberculosis (Table 1).

**Table 1:** Characteristics of tuberculosis patients starting tuberculosis treatment and attending support group meetings, southern India, 2019

Characteristic	No. (%)	
	Patients starting treatment (*n* = 30 706)	Patients attending meetings (*n* = 3651)
**District tuberculosis office**		
Bellary	3 610 (11.8)	754 (20.9)
Bengaluru Urban	6 603 (21.5)	929 (14.1)
Hyderabad	15 177 (49.4)	1564 (10.3)
Koppal	2 174 (7.1)	199 (9.2)
Warangal Urban	3 142 (10.2)	205 (6.5)
**Age, years^a^**		
0–14	1 758 (5.7)	216 (12.3)
15–59	24 587 (80.1)	2985 (12.1)
≥ 60	4 346 (14.2)	449 (10.3)
**Sex**		
Female	12 920 (42.1)	1512 (11.7)
Male	17 786 (57.9)	2139 (12.0)
**Type of case**		
New patient	26 733 (87.1)	3033 (11.3)
Retreatment	2 976 (9.7)	486 (16.3)
Drug-resistant tuberculosis	997 (3.3)	132 (13.2)
**Site of disease**		
Extra-pulmonary	9 719 (31.7)	1005 (10.3)
Pulmonary	20 987 (68.4)	2646 (12.6)
**Diabetes**		
No	28 905 (94.1)	3279 (11.3)
Yes	1 801 (5.9)	372 (20.7)
**HIV infection**		
No	29 560 (96.3)	3557 (12.0)
Yes	1 146 (3.7)	94 (8.2)
**Consumes alcohol regularly **		
No	28 927 (94.2)	3290 (11.4)
Yes	1 779 (5.8)	361 (20.3)
**Participated in peer support group**		
No	27 055 (88.1)	NA
Yes	3 651 (11.9)	NA

HIV: human immunodeficiency virus; NA: not applicable.

^a^ For 15 patients, age was reported as older than 105 years. These data were not considered and age was treated as missing.

### Participation rates

Participation in the meetings varied by district office (Table 1). Participation was higher among retreatment than new patients (16.3%; 486/2876 versus 11.3%; 3033/27 733; *P*-value: < 0.001), patients with diabetes than those without diabetes (20.7%; 372/1801 versus 11.3%; 3279/28 905; *P*-value: < 0.001) and patients who consumed alcohol regularly than those who did not (20.3%; 361/1779 versus 11.4%; 3290/28 927; *P*-value: < 0.001; Table 1). Participation was slightly lower in tuberculosis patients with HIV infection than patients without: 8.2% (94/1146) versus 12.0% (3557/29 560). We did not see any difference in participation by sex and age groups 0–14 years and 15–59 years.

**Fig. 1:**
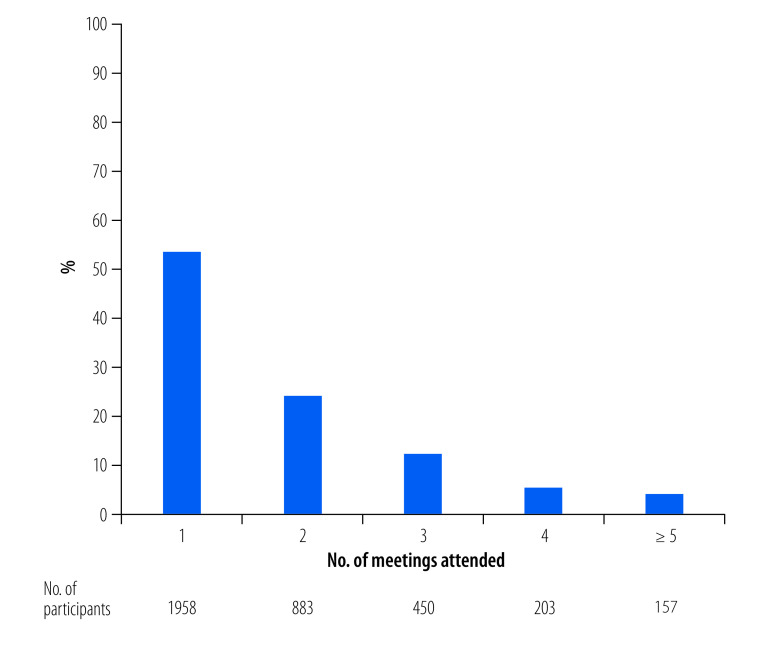
Attendance of tuberculosis patients in support group meetings by number of meetings attended, south India, 2019

Note: In total, 3651 participants attended the meetings.

Of the 3651 patients who attended the meetings, just over half (1958; 53.6%) attended meetings only once and 157 (4.3%) attended five or more meetings (Fig. 1; (available at: https://www.who.int/publications/journals/bulletin/).

### Treatment outcomes

Table 2 shows the tuberculosis treatment outcomes according to participation in meetings. Of the 30 706 tuberculosis patients who started treatment, the treatment outcome was missing for 145, of whom 133 did not attend any meetings; these patients were excluded from our analysis. Of patients who attended at least one support meeting, 94.1% (3426/3639) had a successful treatment outcome while 88.2% (23 745/26 922) of patients who did not attend meetings had a successful treatment outcome. Unsuccessful outcomes, including death, loss to follow-up and not evaluated, were slightly higher in patients who did not attend any meetings than in patients who attended at least one meeting.

**Table 2:** Treatment outcomes of tuberculosis patients by participation in the support group meetings, southern India, 2019

Treatment outcome	No. (%)		
	Did not attend meetings	Attended meetings	Total
Cured	8 531 (31.7)	1906 (52.4)	10 437 (34.2)
Treatment completed	15 214 (56.5)	1520 (41.8)	16 734 (54.8)
**Total with successful outcome**	**23 745 (88.2)**	**3426 (94.1)**	**27 171 (88.9)**
Died	1 512 (5.6)	62 (1.7)	1 574 (5.2)
Lost to follow-up	592 (2.2)	57 (1.6)	649 (2.1)
Treatment failure	152 (0.6)	29 (0.8)	181 (0.6)
Treatment regimen changed	205 (0.8)	27 (0.7)	232 (0.8)
Not evaluated	716 (2.7)	38 (1.0)	754 (2.5)
**Total^a^**	**26 922 (100.0)**	**3639 (100.0)**	**30 561 (100.0)**

^a^ Of the 30 706 tuberculosis patients who started treatment, the treatment outcome was missing for 145, of whom 133 did not attend any meetings; these patients were excluded from our analysis.

Overall, a significantly higher proportion of patients who attended support group meetings (94.1%; 3426/3639) had successful treatment outcomes than patients who did not attend (88.2%; 23 745/26 922; *P*-value: < 0.001; Table 3). The difference in successful treatment outcome by participation in meetings was higher in Bengaluru Urban district than in other districts. Similarly, the higher proportion with a successful treatment outcome in patients who participated in meetings persisted across different characteristics, even for patients with certain risk factors including age ≥ 60 years, drug-resistant tuberculosis, HIV infection, diabetes and regular alcohol consumption (Table 3). Overall, tuberculosis patients who attended support group meetings were two times more likely to have a successful treatment outcome than non-participating patients (unadjusted OR: 2.15; 95% CI: 1.86–2.48), and higher odds of a successful outcome were seen irrespective of patient characteristics (Table 3).

**Table 3:** Successful treatment outcomes in tuberculosis patients by participation in support group meetings and patient characteristics, south India, 2019

Characteristic	Did not attend meetings		Attended meetings		Unadjusted OR (95% CI)
	Total no.	Successful outcome, no. (%)	Total no.	Successful outcome, no. (%)	
**Total**	26 922	23 745 (88.2)	3639	3426 (94.1)	2.15 (1.86–2.48)
**District tuberculosis office**					
Bellary	2 853	2 351 (82.4)	750	689 (91.9)	2.41 (1.82–3.19)
Bengaluru Urban	5 636	4 753 (84.3)	928	886 (95.5)	3.92 (2.85–5.38)
Hyderabad	13 541	12 296 (90.8)	1560	1492 (95.6)	2.22 (1.73–2.85)
Koppal	1 966	1 728 (87.9)	198	180 (90.9)	1.38 (0.83–2.28)
Warangal Urban	2 926	2 617 (89.4)	203	179 (88.2)	0.88 (0.57–1.37)
**Age, in years**					
0–14	1 538	1 433 (93.2)	216	209 (96.8)	2.19 (1.00–4.77)
15–59	21 486	19 206 (89.4)	2973	2805 (94.3)	1.98 (1.69–2.33)
≥ 60	3 885	3 093 (79.6)	449	411 (91.5)	2.77 (1.97–3.90)
**Sex**					
Female	11 346	10 360 (91.3)	1504	1457 (96.9)	2.95 (2.19–3.97)
Male	15 576	13 385 (85.9)	2135	1969 (92.2)	1.94 (1.65–2.29)
**Type of case **					
New patient	23 647	21 107 (89.3)	3032	2893 (95.4)	2.50 (2.10–2.98)
Retreatment	2 486	2 101 (84.5)	486	438 (90.1)	1.67 (1.22–2.30)
Drug-resistant tuberculosis	789	537 (68.1)	121	95 (78.5)	1.71 (1.08–2.71)
**Site of disease**					
Extra-pulmonary	8 670	7 953 (91.7)	1002	972 (97.0)	2.92 (2.02–4.23)
Pulmonary	18 252	15 792 (86.5)	2637	2454 (93.1)	2.09 (1.79–2.44)
**Diabetes **					
No	25 505	22 537 (88.4)	3269	3078 (94.2)	2.12 (1.82–2.47)
Yes	1 417	1 208 (85.2)	370	348 (94.0)	2.74 (1.74–4.31)
**HIV infection **					
No	25 880	22 956 (88.7)	3546	3341 (94.2)	2.08 (1.79–2.40)
Yes	1 042	789 (75.7)	93	85 (91.4)	3.41 (1.63–7.13)
**Regular alcohol consumption **					
No	25 513	22 600 (88.6)	3278	3105 (94.7)	2.31 (1.98–2.71)
Yes	1 409	1 145 (81.3)	361	321 (88.9)	1.85 (1.30–2.64)

CI: confidence intervals; HIV: human immunodeficiency virus; OR: odds ratio.

In the multivariate logistic regression analysis, even after controlling for the potential risk factors, the likelihood of a successful treatment outcome was significantly higher in the patients who attended the support group meetings (Table 4). Overall, after controlling for confounding, the odds of experiencing a successful outcome were 2.4 times greater for the patients who attended the meetings than non-participating patients (aOR: 2.44; 95% CI: 2.10–2.82). The association between participation in meetings and successful treatment outcome was significant only in the districts of Bengaluru Urban (aOR: 4.32; 95% CI: 3.13–5.95), Bellary (aOR: 2.31; 95% CI: 1.74–3.07) and Hyderabad (aOR: 2.31; 95% CI: 1.80–2.98). We found significant associations between participation in meetings and successful treatment outcome for all other patient characteristics, including: age ≥ 60 years (aOR: 3.19; 95% CI: 2.26–4.51); female sex (aOR: 3.33; 95% CI: 2.46–4.50); being a new patient (aOR: 2.76; 95% CI: 2.31–3.30); having extra-pulmonary tuberculosis (aOR: 3.14; 95% CI: 2.16–4.56); having diabetes (aOR: 3.03; 95% CI: 1.91–4.81); HIV infection (aOR: 3.73; 95% CI: 1.76–7.93); and non-consumption of alcohol (aOR: 2.58; 95% CI: 2.19–3.03). Although the observed odds of a successful outcome were slightly lower, we observed that participation in meetings was beneficial to other categories of patients, including patients with drug-resistant tuberculosis (aOR: 1.93; 95% CI: 1.21–3.09) and patients who consumed alcohol regularly (aOR: 1.78; 95% CI: 1.24–2.56).

**Table 4:** Factors associated with successful treatment outcomes in tuberculosis patients participating in support group meetings, south India, 2019

Variable	Successful treatment outcome, aOR (95% CI)^a^
**Overall: meeting participation versus non-participation**	2.44 (2.10–2.82)
**District tuberculosis office **	
Bellary	2.31 (1.74–3.07)
Bengaluru Urban	4.32 (3.13–5.95)
Hyderabad	2.31 (1.80–2.98)
Koppal	1.24 (0.74–2.07)
Warangal Urban	0.94 (0.78–1.13)
**Age, in years**	
0–14	2.39 (1.09–5.22)
15–59	2.28 (1.93–2.69)
≥ 60	3.19 (2.26–4.51)
**Sex**	
Female	3.33 (2.46–4.50)
Male	1.39 (1.28–1.51)
**Type of case**	
New patient	2.76 (2.31–3.30)
Retreatment	1.69 (1.22–2.33)
Drug-resistant tuberculosis	1.93 (1.21–3.09)
**Site of disease**	
Extra-pulmonary	3.14 (2.16–4.56)
Pulmonary	2.31 (1.97–2.72)
**Diabetes **	
No	2.37 (2.03–2.77)
Yes	3.03 (1.91–4.81)
**HIV infection**	
No	2.39 (2.06–2.78)
Yes	3.73 (1.76–7.93)
**Regular alcohol consumption**	
No	2.58 (2.19–3.03)
Yes	1.78 (1.24–2.56)

CI: confidence intervals; HIV: human immunodeficiency virus; OR: odds ratio.

^a^ Adjusted for all the other variables.

## Discussion

Participation of tuberculosis patients in support groups while on treatment significantly improved treatment outcomes. This finding persisted even in patients with a higher risk of unsuccessful outcomes, including patients with drug-resistant tuberculosis, HIV infection and diabetes, and regular alcohol consumers. Only one previous qualitative study in Jharkhand, India reported that patient charter meetings empowered tuberculosis patients and enhanced mental and emotional support from their peers and family members.^20^

The patient-centred approach, involvement of family members, use of facilities that provided privacy to hold the meetings in, inclusion of tuberculosis programme staff, and the designation of a fixed day and time for the meetings were unique aspects of this implementation research. The meetings created a forum that improved communication and understanding between care providers, patients and their family members. Participation of former tuberculosis patients who had successfully completed treatment in the meetings gave the new patients and their family members confidence about the treatment. Patients and their family members were encouraged to share positive experiences related to treatment. Patients were also encouraged to share their experience of any violation of rights or if they had faced any discrimination or denial of rights within the family, community or facility settings. Occasionally, participants provided information on other individuals with symptoms of tuberculosis living in their own homes or in the community. Thus, this forum also led to the identification of new tuberculosis patients. However, we were not able to estimate the number of tuberculosis patients detected through the attendees of the support group meetings, as this information was not recorded clearly. Box 1 gives a summary of the lessons learnt from implementation of the support groups and their achievements. These lessons can be used for future scaling up and strengthening of the programme.

Box 1 **Achievements of and lessons learnt from implementation of support group meetings for tuberculosis patients, southern India, 2019**

**Formation of support groups**

It is feasible to form support groups of active tuberculosis patients, former patients who have been cured or have completed treatment, family members or caregivers, and staff of tuberculosis programmes. Fixing a specific day and time for meetings in a health facility is more convenient for participants to attend. Small group size (15–20 participants) increases people’s involvement during the meeting, even when the group is heterogeneous.

**Capacity-building**

Adequate numbers of tuberculosis field staff need to be trained to ensure support group meetings are held regularly. Developing appropriate action points at the end of each meeting in an interactive manner is useful to discuss unanswered issues.

**Promotion of the meeting**

Tuberculosis programme staff can encourage cured tuberculosis patients to become tuberculosis champions. These former patients attending meetings can convey the benefits of participation to others with tuberculosis symptoms in the family and community, leading to increased detection of tuberculosis cases.

**Time efficiency**

These meetings are also a forum for programme staff to provide important common information to a group of patients because they may not have time to explain this information to each patient individually.

**Community support**

The success of meetings largely depends on community support. These meetings can be used as a way to obtain the support of local donors for the provision of food items for the nutritional requirements of patients in need. Indirectly, this support could reduce stigma associated with tuberculosis in the community.

**Data**

The existing tuberculosis programme data (Nikshay data) can be used to measure the outcomes of meetings. In addition to the existing data, the attendance data of the individual patients and the various activities conducted need to be documented to monitor the programme.

**Achievements**

Participation in the meetings improved tuberculosis treatment outcomes, irrespective of the risk characteristics of the patient.

**Advantages**

No additional staff were needed to conduct the meetings. Existing tuberculosis staff can efficiently facilitate the meetings with appropriate training and mentoring. The cost of organizing the meeting can also be reduced by using local venues and resources, such as furniture and refreshments, from the community and the health facility.

**Research**

Research on support group meetings can be undertaken in different health facilities to determine the best ways to overcome the challenges and to gain community support. Similarly, the perceived benefits felt by patients could be explored using qualitative methods.

The support meetings did not include tuberculosis patients who lived in an institutional setting, such as prisons or homeless shelters, or who did not have family support or a caregiver. Our groups were mixed so gender-specific support could not be provided, particularly for women and adolescent girls with tuberculosis whose needs may differ from men and boys. However, the success of peer support appears to be because of the non-hierarchical reciprocal relationship that is created through the sharing of similar life experiences. This approach of community ownership and mobilization is set out in the national strategic plan on tuberculosis and emphasizes the need for involvement of patient networks in planning, implementation and monitoring of the programme through tuberculosis champions.^2^^,^^3^ Many vocal and confident individuals were identified from these support groups to be trained subsequently as tuberculosis champions. Other studies have shown that peer support approaches are beneficial to patients with other disease conditions.^6^^–^^11^^,^^21^^,^^22^ Numerous studies have reported that peer support through education, training, self-help groups and clubs improves treatment completion for latent and active tuberculosis.^13^^–^^16^^,^^23^^,^^24^ However, these studies do not report a systematic and standard approach for organizing support group meetings among active tuberculosis patients to improve treatment outcomes. Our study provides information on the process for implementation of support group meetings within a health facility. We did not assess the cost and cost–effectiveness of the support group meetings because the CHWs perform many different activities^17^ and it was not feasible to allocate time spent on this specific activity individually. In addition, we did not estimate the costs of training CHWs and providing materials. However, the cost of conducting a meeting was low as the venue was a public health facility and local donors provided the refreshments.

The coronavirus disease 2019 pandemic hindered implementation of the project. With the nationwide lockdown for 21 days on 24 March 2020 and conditional relaxations introduced later, we had to organize virtual support groups using WhatsApp and other virtual platforms. However, this required that at least one member of the household had a smartphone. Fortunately, since the duration of treatment is 6 months, many of the patients who began treatment in 2019 had completed their treatment course before the lockdown.

Our findings have other implications. The outcomes of support group meetings differed between districts. These differences could be because of compromised quality of the support group meetings, the larger size of the groups and the varying levels of successful treatment outcomes within these districts. Nonetheless, even patients with drug-resistant tuberculosis and HIV coinfection who participated in meetings had a greater likelihood of successful treatment outcomes than those who did not participate. Although we did not collect any feedback directly from the participants on the benefits they perceived of attending the meetings, we conducted a process evaluation study using qualitative methods with different stakeholders.^25^

A limitation of our study is that only a small proportion of all tuberculosis patients in the districts participated in these meetings, as the meetings were conducted in urban slum settings only. To achieve a greater impact at a population level, access to these meetings needs to be expanded to increase participation rates. The association of participation in meetings with successful treatment outcomes could have been shaped not only by participation in the intervention itself but also by contextual factors outside of the control of participants and tuberculosis programme staff. For example, the provision of tuberculosis treatment services could vary between different health facilities. In our analysis, we used the data of both tuberculosis patients who attended the meetings and those who did not, thus controlling for this bias to some extent. We did not document many of the activities conducted during the meetings so we could not explore the influence of specific activities on the successful outcome. For example, during the meetings, we obtained the support of local donors for the distribution of food items for needy patients but we did not record this support in a separate format. Getting the doctors from the health facilities with the tuberculosis units to engage with and participate in these meetings regularly was a challenge. The participation of these doctors in the support group meetings is important to provide the tuberculosis patients with the information they need, which the junior tuberculosis programme staff are not be able to provide, and increase the patients’ confidence in the treatment. Another drawback was the lack of mechanisms to obtain feedback from the various stakeholders to understand the unresolved issues during the meetings for continuous improvement. 

Our research provides useful lessons for the implementation of support group meetings at the national level. This approach can be regularized through existing programme staff but would require time and commitment to conduct the meetings regularly. Recently, the Central Tuberculosis Division of the government of India endorsed support group meetings as one of the person-centred care approaches for improved treatment outcomes in its community engagement guidance document.^26^ Another project funded by the United States Agency for International Development has started implementing support group meetings in four states – Assam, Bihar, Karnataka and Telangana – among selected vulnerable populations.^27^
